# Alexithymia, an impairment of emotional cognitive processing, is a candidate risk factor for carotid artery plaque formation in HIV-infected patients

**DOI:** 10.1186/1758-2652-13-S4-P70

**Published:** 2010-11-08

**Authors:** F Vadini, F Sozio, E Mazzotta, C Agostinone, T Ursini, A Agostinone, G Placido, LM Manzoli, G Parruti

**Affiliations:** 1 Ospedale Civile dello Spirito Santo, Psychoinfectivology Unit, Infectious Disease Unit, Pescara, Italy; 2 Ospedale Civile dello Spirito Santo, Infectious Disease Unit, Pescara, Italy;; 3 Epidemiology and Public Health Department, University of Chieti, Chieti, Italy

## Purpose of the study

Vascular aging is now one major concern in the care of HIV infected patients, as many factors may contribute to its faster progression in comparison with the general population. We investigated several psychological factors including Alexithymia, Type D personality, Mental and Physical Components (MCS and PCS) of Quality of Life (QoL) and Depression in a single Italian HIV cohort, well characterized for traditional cardiovascular (CV) risk factors and intima-media thickness of carotid arteries.

## Methods

HIV infected patients followed at our Institution were consecutively enrolled from February to June, 2010. Carotid Intima-Media Thickness and the presence of plaque(s) were investigated by B-mode ultrasonography. Alexithymia was assessed with the 20-item Toronto-Alexithymia-Scale (TAS-20, positive score ≥ 49), Type D personality with the DS14 Distress Scale, depression symptoms with the Beck Depression Inventory (BDI, positive score ≥ 15) and QoL with the SF12 questionnaire. All statistical analyses were carried out using Stata 9.0 package.

## Summary of results

We enrolled 93 HIV infected patients, 75.3% males, aged 45.4±9.8y (r. 21-69), 65.6% infected through heterosexual (39,8%) or homosexual (25,8%) exposure, 32.3% because of drug abuse or transfusion (2,1%). Coinfected patients were 29.0%, smokers 69.8%. As to HAART, 12.9% of patients were untreated, 51.6% on a PI-based and 35.5% on a NNRTI-based regimen, 67.7% of patients with undetectable HIV RNA. Carotid plaques (CP) were found in 40.9% of patients, in 15.0% bilaterally. Patients with CP were significantly older (50.6±8.7 vs 41.8±8.9, p<.0001) and with lower nadir CD4 counts (p=.04); CP were more frequent in hypertensive (p=.02) and lipodystrophic (p=.02) patients. Non significant differences were found between sexes (p=.8), as well as in smokers (p=.08), diabetics (p=.2) and hypercholesterolemic patients (p=.2). Patients with the Alexithymia trait had significantly more CP (p=.002) or bilateral CP (p=.008). Other psychological factors were not associated with CP. Stepwise multivariate logistic regressions confirmed only age (p=.004) and Alexithymia (p=.04) as independently associated with the presence of CP.

## Conclusions

Alexithymia in our series appeared as more tightly associated with CP than most traditional CV risk factors. If this correlation will be confirmed, diagnosis of Alexithymia may allow psychological intervention programs to reduce CV risk in HIV patients.
